# Supplementary material for: Role of epigenetics in unicellular to multicellular transition in Dictyostelium
Source: Genome Biol. 2021 May 4;22:134. doi: 10.1186/s13059-021-02360-9 (PMC8094536; doi:10.1186/s13059-021-02360-9)
Supplement: Supplementary file 1 — Additional file 1: Supplementary figures S1-S15. [file 13059_2021_2360_MOESM1_ESM.docx]

**Role of epigenetics in unicellular to multicellular transition in *Dictyostelium***

**Authors**

Simon Yuan Wang^1,2^, Elizabeth Ann Pollina^3^†, I-Hao Wang^4^†, Lindsay Kristina Pino^5^, Henry L. Bushnell^1,2^, Ken Takashima^1,2^, Colette Fritsche^1^, George Sabin^1^, Benjamin Aaron Garcia^5^, Paul Lieberman Greer^4^, and Eric Lieberman Greer^1,2^ *

**Additional File 1: Figs S1-S15**

Fig. S1

**Fig. S1 Chromatin modifications change during *D. discoideum* transition to multicellularity**

**A,** A decrease in H3K4me3, no change in H3K27ac, and an increase in H3K4me1 is observed by western blot analysis of three biological replicates of *D. discoideum* analyzed at four different stages. Quantification of two independent experiments performed in triplicate is depicted below. *: p<0.05, **: p<0.005, ***: p<0.001, as assessed by multiple comparison one-way ANOVA analysis. **B,** Mass spectrometry analysis shows enrichment of H2AxS1acS8phK15me1 in unicellular stage of *D. discoideum* and enrichment of H3K4me1, H3K14ac, H4K9ac and H4K13acK17me1K21ac in multicellular stages of *D. discoideum*. **C,** Pairwise comparison of histone marks between vegetative stage versus streaming stage, mound stage, fruiting stage, respectively with adjusted p value < 0.05. **D,** Histone modifications of core histones in *D. discoideum*. ac, acetylation; me, methylation; P, phosphorylation. Number of dots under methylated lysines represent mono-, di-, or tri-methylation.

**Fig. S2 ATACseq analysis displays a high degree of reproducibility between replicates of A, vegetative, B, streaming, C, mound, and D, fruiting body stages.**

**Fig. S3 ATACseq analysis of changes in accessible chromatin at different life cycle stages**

**A**, An analysis of chromatin accessibility in relation the nearest TSS demonstrates that unicellular *D. discoideum* have more accessible chromatin further away from the TSS than do multicellular stages. **B**, An analysis of the chromatin accessibility at specific genomic features at different stages reveals that the unicellular stage has a lower degree of accessibility in the promoter and a higher degree of accessibility in exons than multicellular stages.

**Fig. S4 A, Unicellular and B, multicellular stages display unique enriched DNA sequence motifs during ATACseq analysis**

Comprehensive ATACseq motif analysis can be accessed at <https://www.dropbox.com/sh/flf9wqvzz4go7dc/AADr3KXeZQoIr9JjWgyhcikoa?dl=0>.

**
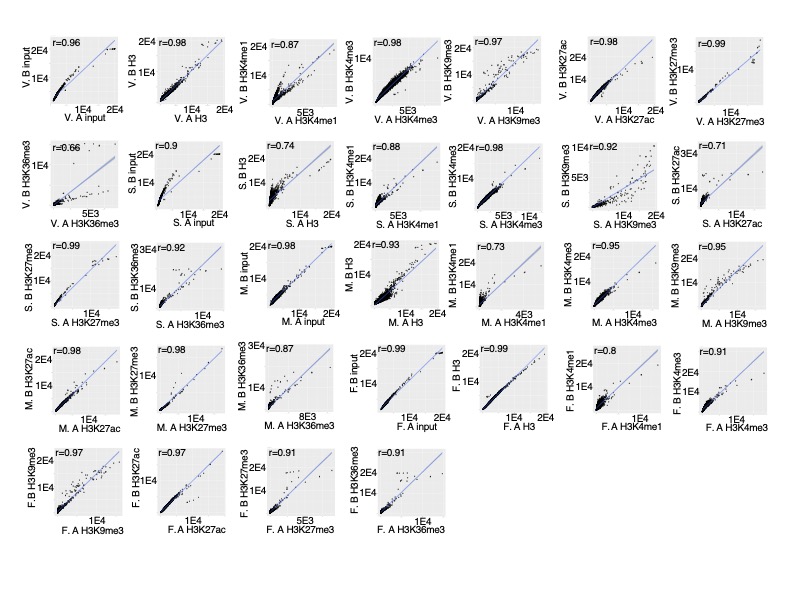
**

**Fig. S5 ChIPseq analysis displays a high degree of reproducibility between replicates of 7 different chromatin immunoprecipitations for vegetative (V), streaming (S), mound (M), and fruiting body (F) stages.**

**Fig. S6 Chromosome distribution, Principal Component analysis, and Feature Distribution of ChIPseq data for 6 chromatin modifications.**

**A,** Chromosome distribution of ChIPseq data for 6 chromatin modifications are displayed across all 6 chromosomes. **B**, PCA of ChIPseq analysis of H3K27me3, H3K9me3, and H3 reveals that these modifications are insufficient to distinguish unicellular from multicellular *D. discoideum*. **C**, An analysis of chromatin modifications distribution at genomic features in the *D. discoideum* genome reveals that chromatin modifications display differences in feature distribution. **D**, H3K36me3 heatmaps did not display distinct patterning in unicellular (vegetative) and multicellular (mound and fruiting body) stages.

**
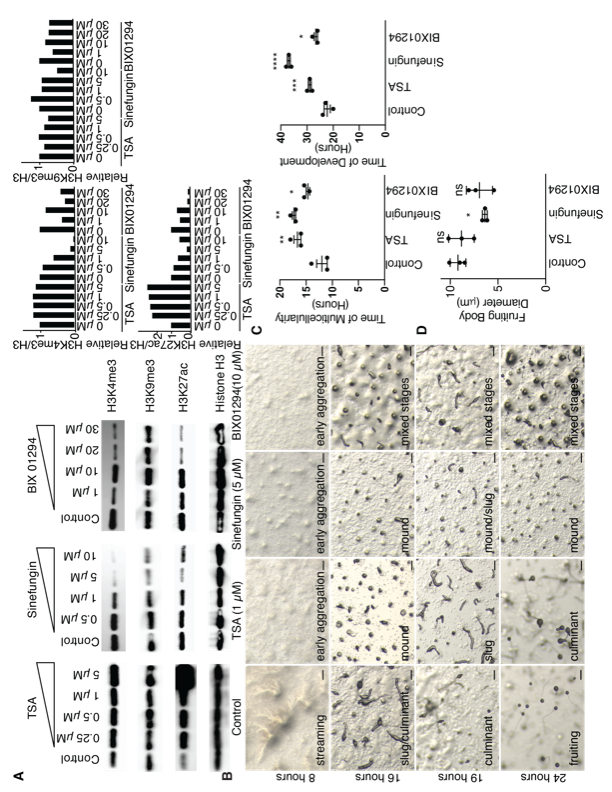
**

**Fig. S7. Chemical inhibitors of chromatin modifications delay *D. discoideum* transition to multicellularity**

**A**, TSA, Sinefungin, and BIX 01294 each display a concentration dependent inhibition of H3K27ac, H3K4me3, H3K9me3 and H3K27ac, and H3K4me3 and H3K27ac respectively as assessed by western blotting in 293T cells. **B**, Representative pictures of *D. discoideum* cellular states on KK2 plates show multicellularity delay after treatment with 5 μM sinefungin, 10 μM BIX 01294, and 1 μM TSA at 8, 16, 19, and 24 hours. Stage is displayed in the lower right hand corner. Scale bars are 20 microns. **C**, Treatment with 5 μM sinefungin, 10 μM BIX 01294, and 1 μM TSA delay *D. discoideum* multicellularity, time to mound stage is shown in graph on left and complete progression to fruiting body stage is shown in graph on right. Each column represents the mean ± the standard error of the mean of three biological replicates performed in triplicate. **: p<0.001, *: p<0.05, ns: not significant, as assessed by one-way ANOVA analysis with Dunnett’s multiple comparisons test. **D**, Treatment with 1 μM TSA and 10 μM BIX 01294 had no effect on *D. discoideum* fruiting body diameter while treatment with 5 μM sinefungin caused a reduction in fruiting body diameter. Fruiting body diameter correlates with the number of cells in each multicellular organism [15, 16]. This graph represents the mean ± the standard error of the mean of three replicates performed with quantification of 5-10 fruiting bodies per replicate. Ns: not significant, *:p<0.05 as assessed by one-way ANOVA analysis with Dunnett’s multiple comparisons test.

**Fig. S8. Chemical inhibitors of chromatin modifications effect on chemotaxis and cell viability**

**A,B**, Treatment with 10 μM BIX 01294 has no effect on *D. discoideum* chemotaxis ability while treatment with 5 μM sinefungin and 1 μM TSA inhibit chemotaxis. Representative images of control cells with or without 250 μM folate (red F on representative pictures) are displayed. The bar graph to the right represents the mean ± the standard error of the mean of three or four biological replicates performed in triplicate. The number of cells which migrated out of the spot where the *D. discoideum* were initially placed was counted in the 30˚ segment towards the 250 μM folate. Ns: not significant, ****:p<0.0001, **:p<0.001 as assessed by one-way ANOVA analysis. Scale bars are 100 microns. **C**, Treatment with 10 μM BIX 01294, 5 μM sinefungin, and 1 μM TSA have no effect on cell death as assessed by trypan blue staining. **D**, Treatment with 10 μM BIX 01294, 5 μM sinefungin, and 1 μM TSA have no effect on cell death as assessed by PI staining. 95°C heat shock for 50 seconds is sufficient to induce cell death. Each column represents the mean ± the standard deviation of three biological replicates. Ns: not significant, ****:p<0.0001 as assessed by one-way ANOVA analysis. **E**, Treatment with 10 μM BIX 01294 and 5 μM sinefungin have no effect on cell death as assessed by annexin V staining while treatment with 1 μM TSA increases annexin V staining. Representative images are displayed on the left while the graph on the right represents the mean ± the standard error of the mean of three biological replicates. Ns: not significant, *:p<0.05 as assessed by t-test.

**
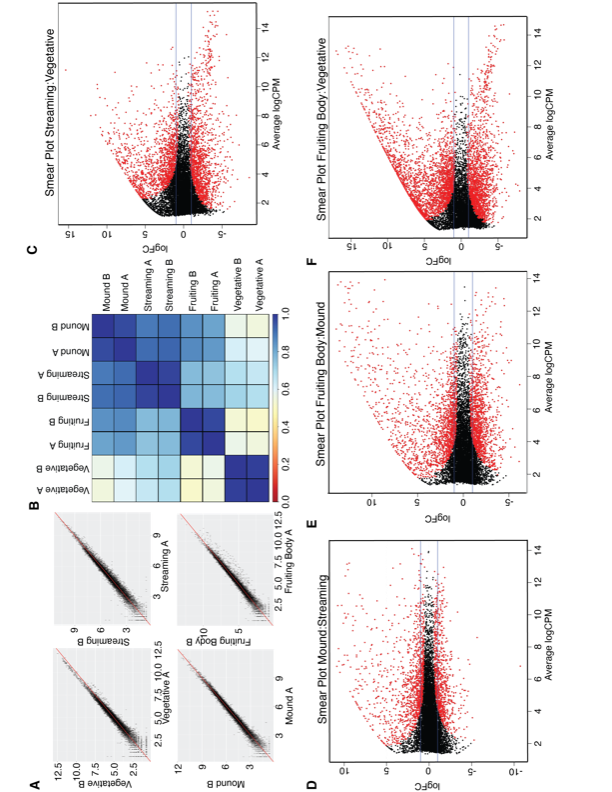
**

Fig. S9

**Fig. S9 RNAseq analysis display a high degree of reproducibility between replicates and can distinguish stages from each other**

**A**, A linear correlation between biological replicates is shown for each stage. r^2^ > 0.95 for each replicate. **B**, A heat map correlation of different RNAseq samples demonstrates the high degree of reproducibility and the differences between the different stages as well as unicellular compared to multicellular stages. **C-F,** A Bland-Altman plot of RNAseq datasets reveals genes which are upregulated and downregulated between individual stages of *D. discoideum*. Red dots represent significant differentially expressed genes, black dots represent not significant expressed genes. **G-J**, Comparison of bulk RNAseq expression to previously published RNAseq data [36] reveals a high degree of correlation.

**Fig. S10 Heat maps of A, transcription factors and B, chromatin modifying enzymes reveal stage specific expression patterns of several global regulators by RNAseq analysis. C, MADS box binding motif is significantly enriched in multicellular enriched expressed genes relative to its occurrence in the genome and unicellular enriched expressed genes. P values were calculated by hypergeometric probability.**


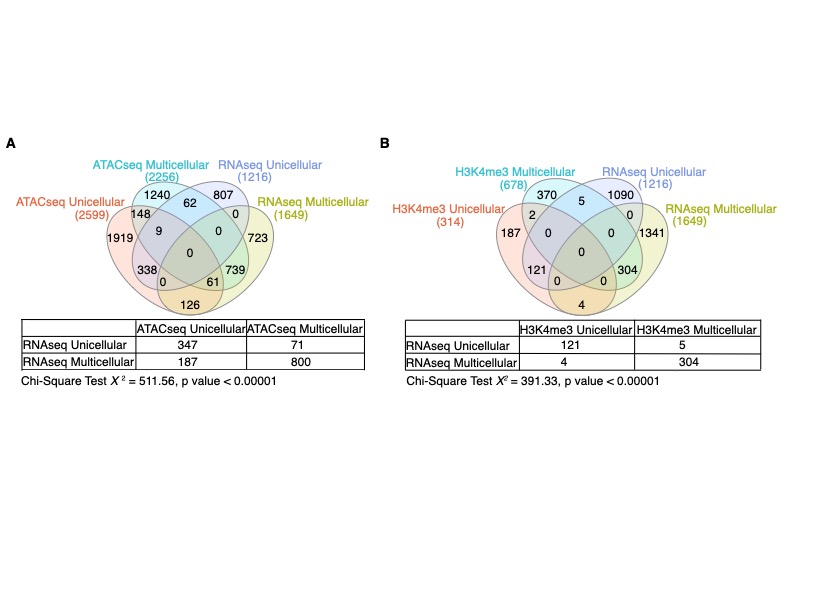


**Fig. S11 Comparison of ATACseq and H3K4me3 ChIPseq to unicellular and multicellular enriched genes**

**A**, Venn diagram shows the overlap of significant unicellular genes identified by RNAseq are enriched for greater accessibility in unicellular ATACseq and multicellular genes identified by RNAseq are enriched for greater accessibility in multicellular ATACseq as assessed by chi-squared test, p<0.05. **B**, Venn diagram shows the overlap of significant unicellular genes identified by RNAseq are enriched for H3K4me3 in unicellular ChIPseq while multicellular genes identified by RNAseq are enriched for H3K4me3 in multicellular ChIPseq as assessed by chi-squared test, p<0.05.

**
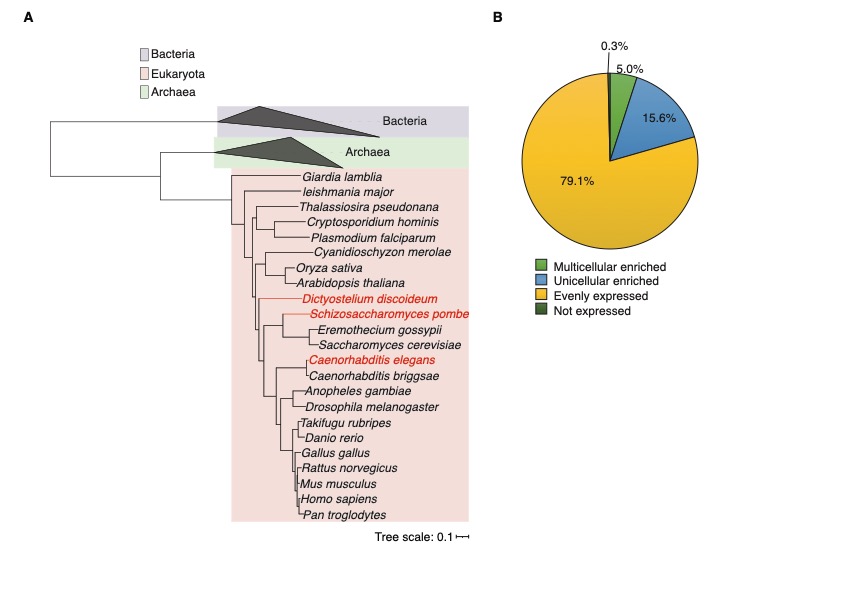
**

**Fig. S12 Orthologue analysis of genes in *D. discoideum, S. pombe,* and *C. elegans* reveals unicellular or multicellular enrichment**

**A**, Phylogenetic tree of *D. discoideum, S. pombe,* and *C. elegans* and other unicellular and multicellular eukaryotes. Scale bar of 0.1 represents the length of the branch required for a 10% genetic change. **B**, A pie-chart breaks down whether the 2473 conserved genes are expressed in both unicellular and multicellular stages (yellow; 79.1%), enriched expression in the unicellular stage (blue; 15.6%), enriched expression in the multicellular stages (light green; 5%) or not expressed (dark green; 0.3%).

**Fig. S13 Sequencing of deletion strains**

Each mutant strain was validated by sanger sequencing.

**
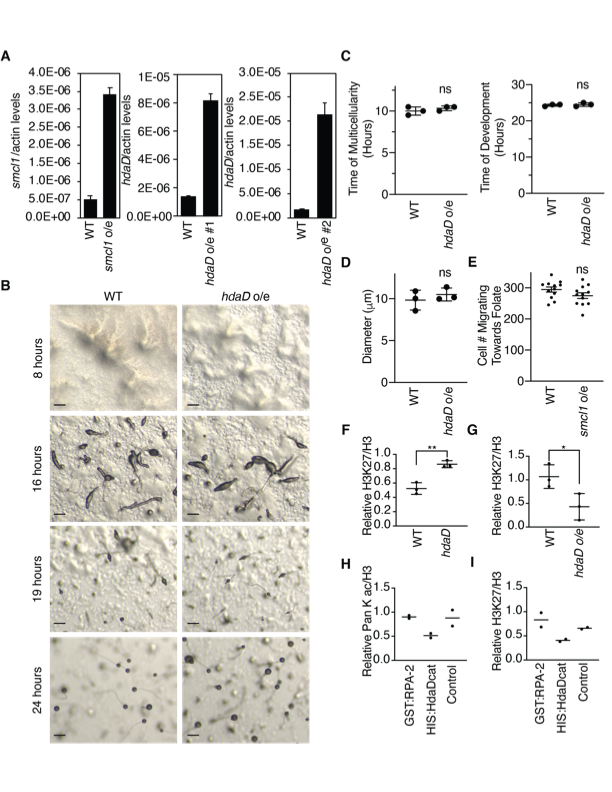
**

**Fig. S14 *hdaD* overexpression has no effect on multicellularity**

**A**, Real time RT PCR confirms overexpression of *smcl1* and *hdaD*. Each bar represents the mean ± the standard deviation of 3 replicates. **B**, Representative images of *hdaD* overexpression reveals similar multicellularity timing to WT *D. discoideum*. Scale bars are 20 microns. **C**, Overexpression of *hdaD* causes no effect on multicellularity (upper graph) and progression to fruiting body stage (lower graph). This graph represents the mean ± the standard error of the mean of three independent biological replicates performed in triplicate. Ns: not significant, as assessed by unpaired t test. **D**, Overexpression of *hdaD* has no effect on the diameter of *D. discoideum* fruiting bodies. This graph represents mean ± the standard error of the mean of three independent biological replicates performed once. Ns: not significant, as assessed by unpaired t test. **E**, Overexpression of *smcl1* had no effect on chemotaxis of *D. discoideum.* Each column represents the mean ± the standard error of the mean of three biological replicates performed in triplicate. The number of cells which migrated towards the 30˚ segment containing 250 μM folate was counted. Ns: not significant, as assessed by unpaired t test. **F-G**, Quantification of western blots shown in Fig. 5F + 5G. Plots represent the mean ± the standard deviation of three biological replicates. *: p<0.05, **: p<0.01 as assessed by t-test. **H**, Quantification of western blots shown in Fig. 5H.

**Fig. S15 Electron microscopy of WT, *hdaD* knockout and overexpression strains and TSA treated cells reveal no difference in nucleolus diameter**

**A**, Representative electron microscopy images of *hdaD* overexpression and TSA treated cells. The scale bars represent 500 nm. Heterochromatin regions are highlighted in red triangles. % heterochromatin was calculated by examining the condensed chromatin which appears darker in the nucleus (outlined in yellow) while excluding the nucleolus (outlined in blue) from calculations. **B**, Quantification of nucleolus diameter in nm. 30 images were measured in each condition. Ns: not significant as assessed by multiple comparison one-way ANOVA analysis.
