# Supplementary material for: Role of epigenetics in unicellular to multicellular transition in Dictyostelium
Source: Genome Biol. 2021 May 4;22:134. doi: 10.1186/s13059-021-02360-9 (PMC8094536; doi:10.1186/s13059-021-02360-9)
Supplement: Supplementary file 10 — Additional file 10. Review history. [file 13059_2021_2360_MOESM10_ESM.docx]

Review History

**First round of review**

**Reviewer 1**

**Are you able to assess all statistics in the manuscript, including the appropriateness of statistical tests used?**

Yes, and I have assessed the statistics in my report.

**Comments to author:**

In this manuscript, Wang et al assessed transcriptomics, epigenomics and chromatin accessibility profiles of Dictyostelium discoideum at unicellular and multicellular stages in order to understand the role of chromatin biology in driving multicellularity. D. discoideum is a rare organism that readily undergoes multicellularity upon nutrition shortage and can revert back to its unicellular state. Through their analyses, authors showed that patterns of H3K4 methylation and H3K27 acetylation undergo significant changes during multicellular state transition. Through orthologous gene analyses, authors also identified gene sets specifically expressed in unicellular or multicellular state of D. discoideum.

D. discoideum is an important model organism and the datasets presented in this study will serve as a great resource to the research community. Following are a few comments that concern the overall clarity and rigor of the data analysis and manuscript.

Major comments

1. Authors discuss a correlation between the different datasets, ChIP-seq, ATAC-seq and RNA-seq, for srfA (Figure 3A,B). In addition to the specific example of srfA, it will be interesting to see if differentially expressed gene (higher in unicellular or in multicellular states) from RNA-seq data analysis significantly overlap with differentially accessible genes identified through ATAC-seq or differentially bound genes from ChIP-seq data.

2. The authors use the drug Sinefungin to determine the impact of H3K4me3 on multi-cellularization. However, they show the drug decreases both H3K4me3 and H3K9me3. These marks have very different biological significance. A decrease in H3K9me3 is expected to lead to a derepression of centrometric and telomeric heterochromatin, and thus transcriptional activation of repeats. This could, in and of itself, lead to cell cycle delays that could explain some of the observed phenotypes. Thus, the authors need to use a more specific drug or strongly tone down conclusions regarding H3K4me3 and explain the caveats in the text.

3. The authors investigate the role of hdaD in regulating the transition from unicellularity to multicellularity by using an overexpression strain. Although a putative histone deacetylase seems like a plausible candidate to drive the shift from unicellularity to multicellularity, hdaD knockout presented with the least significant phenotypes related to multicellularity. In order to confirm the role of hdaD in driving multicellularity in D. discoideum, authors should do additional experiments: (i) either assess the epigenomic landscape and chromatin accessibility in hdaD transgenics and/or (ii) generate and characterize a deacetylase dead-hdaD transgenics.

4. For general reproducibility of	code	and	analyses, all	analytical	code	for	this	study	should be deposited in a repository such Github or made available as a supplemental file. In addition, since software versions can strongly impact results, the authors should systematically provide in the methods the version number of all used standalone software (e.g. R, bowtie2, fastqc, MACS2, Homer, etc.), packages (e.g. edgeR, clusterprofiler, diffbind, Chipseeker, etc.) and database (e.g. GO term database, genome version).

5. For all Western blot analyses, please provide the data from all performed replicates (not just a representative panel), as well as a quantification, for instance using ImageJ. This includes the deacetylation experiment from Figure 5I, but also drug impact experiments, etc.

Minor comments

1. It is very interesting that different knockout mutants present with varying phenotypes in terms of transition of multicellular state. For example, srfA is slowed in both time of multicellularity and development, whereas smcl1 never reaches multicellularity. Relative to the srfA deletion mutant, ammr1 knockout mutant show more delayed time of multicellularity and development (Figure 4B). However, it's fruiting body diameter and cell # migrating towards folate are more similar to wt controls than the srfA deletion mutant (Figure 4C). Additionally, overexpression of smcl1 delayed the time of multicellularity and development, while resulting in increased fruiting diameter and cell # migrating towards folate. As authors are introducing D. discoideum as a model organism to study the evolution of multicellularity, relevance/possible applications of each mutant strains as a model should be discussed. Some questions that should be explicitly discussed are: What does it mean to be delayed in reaching multicellularity? Would delayed time to multicellularity/development affect the survival of the organism? What effects could an increased fruiting body diameter have on the organism? Additionally, a time curve that tracks phenotypic changes of the knockout mutants and o/e strains could be a great resource to the research community.

2. The methods state that both input and total H3 were used to normalize ChIP-seq data sets of histone modifications. However, which analysis used which control is unclear. In addition, whether changes in H3 occupancy were observed between the multicellular and unicellular states is also unclear. The authors should make these aspects explicit.

3. The fruiting body diameter of the HspH2 mutant looks increased in the figure 4A - this should be quantified and added to 4C.

4. Global histone H3K4me3 levels seem to decrease upon transition to multicellularity, according to the western blot data presented in Figure S1, which cannot be measured by ChIP-seq data (as in Figure 1G) without a spike-in control. It would be important for the authors to discuss this caveat. This is especially important as H3K4me3 is a key factor in separating unicellular and multicellular states (Figure 1F).

5. It is not clear which gene/peak sets are used for different panels. For example, which gene set is used for the heatmap of ATAC-seq peaks in Figure 1B? ChIP-seq peaks in Figure 1G? Characterization/evaluation of different gene sets used for specific analyses needs to be explicit.

6. For Figure 3H, it will be interesting to check if expression of unicellular state-specific gene, hspH2, is vegetative state-biased as well.

7. In lines 31 ("delete 4 additional …") and 48 ("Deletion of all for …") of page 16, the text may be construed to indicate deletion of multiple genes. Authors should modify the sentence to clearly state that these are single deletion mutants.

8. Authors may consider adding Annexin V staining (as shown in Figure S8E) for the viability analysis Figure 4E, as it seems to be more sensitive to differences in cell viability than PI staining.

**Reviewer 2**

**Are you able to assess all statistics in the manuscript, including the appropriateness of statistical tests used?**

Yes, and I have assessed the statistics in my report.

**Comments to author:**

I greatly enjoyed reading Wang et al.'s research on the epigenetic basis underlying the transition to multicellularity in the social amoeba Dictyostelium discoideum. This work is a monumental piece of scientific investigation that integrates methods and concepts from several fields of investigation, namely evolutionary biology, epigenetics and cell biology. I congratulate the authors for their work. The authors show how specific histone modifications are key to transition from the vegetative state to the social one. These modifications are first identified through ATAC-Seq and ChipSeq, and then their necessity for the timing of this key transition is tested pharmacologically. Transcriptomics is used to identify what genetic targets are differentially expressed in vegetative vs. multicellular D. discoideum colonies, highlighting once more the key role of histone modifiers in association with either state. Using a comparative biology approach, which focuses on finding molecular signatures of unicellular or multicellular states between either D. discoideum states and either uni- or multi-cellular organisms, helped reveal key target genes - including ribosomal protein genes - that may be importantly associated with either cell organization state. What sets this work beyond an exploratory association between gene expression/activation and either uni- or multi-cellular state, is the functional validation of the sufficiency of genes associated with multicellularity. In this part, the authors prove that knocking-out four such genes has dramatic effects on the initiation/dynamics of transition to multicellularity.

Like I expressed already, I am a big fan of this work and I have no concerns over this work.

If there is something that would be useful/important to observe, here are a few suggestions to strengthen the manuscript: is 1. a phylogenetic tree placing D. discoideum in relation with other organisms that evolved multi-cellularity. 2. a phylogenetic tree built on the genes associated with multicellularity.

It would be interesting to know more about whether genes associated with multicellularity underwent neofunctionalization or acquired novel domains in the transition from uni-cellular to multicellular organims. However, I do acknowledge it may be complicated to answer this question as the evolution of D. discoideum dates too far back in time. In this case, are there closely related species to D. discoideum that either present or lack the uni- and multi-cellular strategies? If yes, is there conservation in genes associated with the transition to multicellularity?

**Authors Response**

**Point-by-point responses to the reviewers’ comments:**

*We would like to thank all of the reviewers and editor for volunteering their time and effort to provide ideas for enhancing our manuscript. We appreciate their insightful comments and enthusiasm for this manuscript. To address the reviewers’ points, we have now included several new experiments and have modified our manuscript accordingly. We feel that our study has significantly improved as a result of these changes, and would like to thank the reviewers once again for their helpful suggestions. Below we have listed the new or updated figure panels and then provide a point-by-point response to each of the reviewers’ comments.*

*List of new or updated figure panels:*

*Figure 3H. we have updated this figure to include the expression pattern of hspH2 in the single cell RNAseq data.*

*Figure 4C. we have updated this to include the fruiting body diameter of hspH2 knockouts*

*New Figures 5J+I. electron microscopy images and quantification of WT and hdaD knockout dictyostelium reveals that hdaD KO causes a decrease in heterochromatin similar to TSA treatment.*

*Modified Supplementary Figure 7A. We have included on the right the quantification of western blots after chemical treatment of Dictyostelium.*

*New Supplementary Figure 11. Comparison of ATACseq and H3K4me3 ChIPseq to unicellular and multicellular gene expression*

*New supplementary Figure 12A. We have included a phylogenetic tree to display D. discoideum relative to S. pombe and C. elegans and other unicellular and multicellular eukaryotes.*

*We moved Figure 5F to Supplementary Figure 14E.*

*New Supplementary Figures 14F-I. Quantifications of western blots in Figures 5F-H.*

*New Supplementary Figure 15. Representative EM images of hdaD overexpression and quantification of nucleolus diameters of EM images to demonstrate that comparisons were done at equivalent cells.*

Reviewer #1: In this manuscript, Wang et al assessed transcriptomics, epigenomics and chromatin accessibility profiles of Dictyostelium discoideum at unicellular and multicellular stages in order to understand the role of chromatin biology in driving multicellularity. D. discoideum is a rare organism that readily undergoes multicellularity upon nutrition shortage and can revert back to its unicellular state. Through their analyses, authors showed that patterns of H3K4 methylation and H3K27 acetylation undergo significant changes during multicellular state transition. Through orthologous gene analyses, authors also identified gene sets specifically expressed in unicellular or multicellular state of D. discoideum.

D. discoideum is an important model organism and the datasets presented in this study will serve as a great resource to the research community. Following are a few comments that concern the overall clarity and rigor of the data analysis and manuscript.

*We thank the reviewer for their overall enthusiasm for our manuscript and for their suggestions to improve the manuscript. We have addressed all the comments with new experiments or altered text.*

Major comments

1. Authors discuss a correlation between the different datasets, ChIP-seq, ATAC-seq and RNA-seq, for srfA (Figure 3A,B). In addition to the specific example of srfA, it will be interesting to see if differentially expressed gene (higher in unicellular or in multicellular states) from RNA-seq data analysis significantly overlap with differentially accessible genes identified through ATAC-seq or differentially bound genes from ChIP-seq data.

*In Figure 3A we do show that the genes which have higher expression from RNAseq data sets at each stage are more accessible by ATACseq. Per the reviewers suggestion, we have now increased this analysis to examine the unicellular or multicellular enriched expressed genes chromatin accessibility by ATACseq and marking by H3K4me3 as assessed by ChIPseq in new supplementary figure 11.*

2. The authors use the drug Sinefungin to determine the impact of H3K4me3 on multi-cellularization. However, they show the drug decreases both H3K4me3 and H3K9me3. These marks have very different biological significance. A decrease in H3K9me3 is expected to lead to a derepression of centrometric and telomeric heterochromatin, and thus transcriptional activation of repeats. This could, in and of itself, lead to cell cycle delays that could explain some of the observed phenotypes. Thus, the authors need to use a more specific drug or strongly tone down conclusions regarding H3K4me3 and explain the caveats in the text.

*We thank the reviewer for catching this. We have clarified the language as suggested.*

3. The authors investigate the role of hdaD in regulating the transition from unicellularity to multicellularity by using an overexpression strain. Although a putative histone deacetylase seems like a plausible candidate to drive the shift from unicellularity to multicellularity, hdaD knockout presented with the least significant phenotypes related to multicellularity. In order to confirm the role of hdaD in driving multicellularity in D. discoideum, authors should do additional experiments: (i) either assess the epigenomic landscape and chromatin accessibility in hdaD transgenics and/or (ii) generate and characterize a deacetylase dead-hdaD transgenics.

*We thank the reviewer for their suggestion and have now performed electron microscopy to further demonstrate that the histone deacetylase that we characterized in this manuscript has an effect on chromatin landscape. We found that hdaD deletion, similarly to TSA treatment, causes a decrease in heterochromatin. We have included this data in new Figs. 5I, 5J and new supplementary figure 15.*

4. For general reproducibility of code and analyses, all analytical code for this study should be deposited in a repository such Github or made available as a supplemental file. In addition, since software versions can strongly impact results, the authors should systematically provide in the methods the version number of all used standalone software (e.g. R, bowtie2, fastqc, MACS2, Homer, etc.), packages (e.g. edgeR, clusterprofiler, diffbind, Chipseeker, etc.) and database (e.g. GO term database, genome version).

*We have deposited all the codes for sequencing in GitHub https://github.com/BCHGreerlab/Dictyostelium . The version numbers of all software have been added in the manuscript.*

5. For all Western blot analyses, please provide the data from all performed replicates (not just a representative panel), as well as a quantification, for instance using ImageJ. This includes the deacetylation experiment from Figure 5I, but also drug impact experiments, etc.

*We have added all the uncropped western images in the supplementary file and uploaded all these raw images and quantifications to Mendeley data https://data.mendeley.com/datasets/zfhh6xzck4/draft?a=7b9f086c-751e-4a4c-8cf6-813e9f0d4ff1 . We have also included quantifications of all western blots in new supplementary figures 7A and 14F-I.*

Minor comments

1. It is very interesting that different knockout mutants present with varying phenotypes in terms of transition of multicellular state. For example, srfA is slowed in both time of multicellularity and development, whereas smcl1 never reaches multicellularity. Relative to the srfA deletion mutant, ammr1 knockout mutant show more delayed time of multicellularity and development (Figure 4B). However, it's fruiting body diameter and cell # migrating towards folate are more similar to wt controls than the srfA deletion mutant (Figure 4C). Additionally, overexpression of smcl1 delayed the time of multicellularity and development, while resulting in increased fruiting diameter and cell # migrating towards folate. As authors are introducing D. discoideum as a model organism to study the evolution of multicellularity, relevance/possible applications of each mutant strains as a model should be discussed. Some questions that should be explicitly discussed are: What does it mean to be delayed in reaching multicellularity? Would delayed time to multicellularity/development affect the survival of the organism? What effects could an increased fruiting body diameter have on the organism? Additionally, a time curve that tracks phenotypic changes of the knockout mutants and o/e strains could be a great resource to the research community.

*We thank the reviewer for reiterating some of the exciting questions which our study raises for the field of evolution in general. We have expanded the discussion as per the reviewers suggestion. We agree that producing time curves that track each of the phenotypic changes in the knockout and o/e strains would be a great resource but due to equipment limitations we could not produce those for this study. We think that this will be an interesting resource to produce in follow up work after acquiring equipment which can produce time-lapse videos throughout the entire phenotypic time course.*

2. The methods state that both input and total H3 were used to normalize ChIP-seq data sets of histone modifications. However, which analysis used which control is unclear. In addition, whether changes in H3 occupancy were observed between the multicellular and unicellular states is also unclear. The authors should make these aspects explicit.

*We have further clarified the methods as suggested. We used H3 for the normalization of ChIP-seq data for MACS2 peak calling and also for differential analysis. We showed in figure S6B that H3 ChIPseq, and therefore nucleosome occupancy, was not capable of distinguishing between the different stages in PCA plots. We have further highlighted this finding in the text. We also found no significantly differentially bound genes in the H3 ChIPseq between different states. We further applied ATACseq to test whether there was any difference in fragment size distribution between multicellular or unicellular stages as an indirect way to estimate the nucleosome allocations and didn’t observe dramatic differences despite the slight enrichment of mononucleosomes in the fruiting stage (Reviewer Fig. 1).*

3. The fruiting body diameter of the HspH2 mutant looks increased in the figure 4A - this should be quantified and added to 4C.

*We have modified the figure and included this result in the text as suggested.*

4. Global histone H3K4me3 levels seem to decrease upon transition to multicellularity, according to the western blot data presented in Figure S1, which cannot be measured by ChIP-seq data (as in Figure 1G) without a spike-in control. It would be important for the authors to discuss this caveat. This is especially important as H3K4me3 is a key factor in separating unicellular and multicellular states (Figure 1F).

*We have included this caveat in the discussion.*

5. It is not clear which gene/peak sets are used for different panels. For example, which gene set is used for the heatmap of ATAC-seq peaks in Figure 1B? ChIP-seq peaks in Figure 1G? Characterization/evaluation of different gene sets used for specific analyses needs to be explicit.

*We have included increased information in the figure legends.*

6. For Figure 3H, it will be interesting to check if expression of unicellular state-specific gene, hspH2, is vegetative state-biased as well.

*Thank you for this suggestion. We have included hspH2 in the updated figure 3H and it does show enrichment in the unicellular cells as well.*

7. In lines 31 ("delete 4 additional …") and 48 ("Deletion of all for …") of page 16, the text may be construed to indicate deletion of multiple genes. Authors should modify the sentence to clearly state that these are single deletion mutants.

*We have clarified this language as suggested.*

8. Authors may consider adding Annexin V staining (as shown in Figure S8E) for the viability analysis Figure 4E, as it seems to be more sensitive to differences in cell viability than PI staining.

*Each stain is used to measure cell death. Since PI is typically used for assessing late stage apoptotic cells we felt this would be most appropriate for distinguishing live vs dead cells.*

Reviewer #2: I greatly enjoyed reading Wang et al.'s research on the epigenetic basis underlying the transition to multicellularity in the social amoeba Dictyostelium discoideum. This work is a monumental piece of scientific investigation that integrates methods and concepts from several fields of investigation, namely evolutionary biology, epigenetics and cell biology. I congratulate the authors for their work. The authors show how specific histone modifications are key to transition from the vegetative state to the social one. These modifications are first identified through ATAC-Seq and ChipSeq, and then their necessity for the timing of this key transition is tested pharmacologically. Transcriptomics is used to identify what genetic targets are differentially expressed in vegetative vs. multicellular D. discoideum colonies, highlighting once more the key role of histone modifiers in association with either state. Using a comparative biology approach, which focuses on finding molecular signatures of unicellular or multicellular states between either D. discoideum states and either uni- or multi-cellular organisms, helped reveal key target genes - including ribosomal protein genes - that may be importantly associated with either cell organization state. What sets this work beyond an exploratory association between gene expression/activation and either uni- or multi-cellular state, is the functional validation of the sufficiency of genes associated with multicellularity. In this part, the authors prove that knocking-out four such genes has dramatic effects on the initiation/dynamics of transition to multicellularity.

*We would like to thank the reviewer for their excitement about our work!*

Like I expressed already, I am a big fan of this work and I have no concerns over this work.

If there is something that would be useful/important to observe, here are a few suggestions to strengthen the manuscript: is 1. a phylogenetic tree placing D. discoideum in relation with other organisms that evolved multi-cellularity. 2. a phylogenetic tree built on the genes associated with multicellularity.

*We thank the reviewer again for their enthusiasm about our work! We have included a phylogenetic tree as suggested by the reviewer in new supplementary figure 12A.*

It would be interesting to know more about whether genes associated with multicellularity underwent neofunctionalization or acquired novel domains in the transition from uni-cellular to multicellular organims. However, I do acknowledge it may be complicated to answer this question as the evolution of D. discoideum dates too far back in time. In this case, are there closely related species to D. discoideum that either present or lack the uni- and multi-cellular strategies? If yes, is there conservation in genes associated with the transition to multicellularity?

*We agree that these are exceptionally interesting questions. We think that an epigenetic examination of some of the unicellular amoebozoa which are phylogenetically closer to D. discoideum will yield a more refined mapping of the importance of epigenetics in this process. Interestingly, of the ~120 known Dictyostelia, all of them have the capacity to transition to multicellularity, but the timing of this can differ, as can the dependency on cyclic adenosine monophosphate or the presence of a stalk. We agree that these are exciting questions and think that a deeper examination of different Dictyostelia or other Amoebozoa which cannot transition to multicellular forms would be revealing. Many of the unicellular amoebozoa use cAMP only as an intracellular secondary messenger, but the group that contains D. discoideum, secrete cAMP and use this to coordinate the cellular aggregation. This difference in cell to cell communication could have been one of the necessary components which facilitated the transition to multicellularity. In follow up studies it will be interesting to perform comparative biology experiments to examine whether secreted cAMP has any cross talk with the epigenetic regulation that helps to regulate multicellularity in D. discoideum. We have included some additional statements in the discussion to highlight these interesting questions.*

**Second round of review**

**Reviewer 1**

The authors have addressed all my major concerns. I believe this manuscript will be a great resource for both the model organism and the study of evolution of multicellularity.

Small note: the version for cutadapt is still missing and should be added in the final manuscript.
